# Duration of antibiotic therapy in critically ill patients: a randomized controlled trial of a clinical and C-reactive protein-based protocol versus an evidence-based best practice strategy without biomarkers

**DOI:** 10.1186/s13054-020-02946-y

**Published:** 2020-06-01

**Authors:** Isabela Borges, Rafael Carneiro, Rafael Bergo, Larissa Martins, Enrico Colosimo, Carolina Oliveira, Saulo Saturnino, Marcus Vinícius Andrade, Cecilia Ravetti, Vandack Nobre, on behalf of NIIMI – Núcleo Interdisciplinar de Investigação em Medicina Intensiva, Isabela N. Borges, Rafael M. Carneiro, Rafael Bergo, Larissa N. Martins, Enrico A. Colosimo, Carolina F. Oliveira, Saulo F. Saturnino, Marcus Vinícius M. Andrade, Cecilia G. Ravetti, Vandack Nobre

**Affiliations:** 1 Graduate Program in Health Sciences: Infectious Diseases and Tropical Medicine, Department of Internal Medicine, School of Medicine and Hospital das Clínicas, Universidade Federal de Minas Gerais, Belo Horizonte, Brazil; 2 Departamento de Clínica Médica, 2° andar Faculdade de Medicina. Av. Alfredo Balena, 190, Santa Efigênia, Belo Horizonte, Minas Gerais Brazil; 3 Graduate Program in Statistics, Department of Statistics, Universidade Federal de Minas Gerais, Belo Horizonte, Brazil; 4 Graduate Program in Health Sciences: Adult Health, Department of Internal Medicine, School of Medicine and Hospital das Clínicas, Universidade Federal de Minas Gerais, Belo Horizonte, Brazil

**Keywords:** Sepsis, Infection, Critical illness, Antibiotic, Antibiotic stewardship, C-reactive protein

## Abstract

**Background:**

The rational use of antibiotics is one of the main strategies to limit the development of bacterial resistance. We therefore sought to evaluate the effectiveness of a C-reactive protein-based protocol in reducing antibiotic treatment time in critically ill patients.

**Methods:**

A randomized, open-label, controlled clinical trial conducted in two intensive care units of a university hospital in Brazil. Critically ill infected adult patients were randomly allocated to (i) intervention to receive antibiotics guided by daily monitoring of CRP levels and (ii) control to receive antibiotics according to the best practices for rational use of antibiotics.

**Results:**

One hundred thirty patients were included in the CRP (*n* = 64) and control (*n* = 66) groups. In the intention-to-treat analysis, the median duration of antibiotic therapy for the index infectious episode was 7.0 (5.0–8.8) days in the CRP and 7.0 (7.0–11.3) days in the control (*p* = 0.011) groups. A significant difference in the treatment time between the two groups was identified in the curve of cumulative suspension of antibiotics, with less exposure in the CRP group only for the index infection episode (*p* = 0.007). In the per protocol analysis, involving 59 patients in each group, the median duration of antibiotic treatment was 6.0 (5.0–8.0) days for the CRP and 7.0 (7.0–10.0) days for the control (*p* = 0.011) groups. There was no between-group difference regarding the total days of antibiotic exposure and antibiotic-free days.

**Conclusions:**

Daily monitoring of CRP levels may allow early interruption of antibiotic therapy in a higher proportion of patients, without an effect on total antibiotic consumption. The clinical and microbiological relevance of this finding remains to be demonstrated.

**Trial registry:**

ClinicalTrials.gov Identifier: NCT02987790. Registered 09 December 2016.

## Background

Increasing concerns about antimicrobial abuse and development of bacterial resistance have fueled the search for the objective and rational use of antibiotics. There is growing evidence supporting the use of shorter antibiotic courses to treat various types of infection, with clinical outcomes similar to those obtained with longer treatments [1–6]. Individualization of antibiotic treatment time has been gaining importance [7–10]. This measure prevents unnecessary exposure to antibiotics while reducing the risk of therapeutic failure in those with a late response.

Circulating inflammatory biomarkers have been used as a guide to support treatment individualization. One useful marker is procalcitonin (PCT), whose benefit in reducing antibiotic treatment time was demonstrated in several studies [11–16], including a potential reduction in the mortality of critically ill patients [14–16]. Nevertheless, the high cost of PCT testing reduces its availability in some settings [10, 17]. In this context, C-reactive protein (CRP) may be a reasonable low-cost alternative [10, 16, 18]. A recent meta-analysis demonstrated that the PCT-guided algorithms only showed a survival benefit when used in combination with CRP, along with other specificities [16]. Nevertheless, few studies testing a CRP-guided strategy have been conducted in adult critical ill patients. A recent single-center clinical trial involving patients with sepsis suggested that CRP may be a useful marker to guide antibiotic treatment time, when compared to a PCT strategy [19].

The objective of this study was to test the impact of a decision flowchart based on CRP serum levels and clinical features on the duration of antibiotic therapy in critically ill infected patients, compared to a control group treated according to the best available evidence for rational antibiotic treatment in this population.

## Methods

### Study design

This was an open-label, randomized, parallel-group trial conducted in two intensive care units (ICUs) of the *Hospital das Clínicas da Universidade Federal de Minas Gerais* (UFMG) between January 2017 and May 2018, in Belo Horizonte, Brazil [20]. The study was enrolled in ClinicalTrials.gov (NCT02987790) and approved by the ethics committee of the home institution.

#### Participants and randomization

All adult patients (age ≥ 18 years) admitted to the ICUs were considered for potential inclusion according to clinical suspicion or microbiological confirmation of infection and the prospect of an ICU stay longer than 24 h. The diagnosis of sepsis or septic shock was considered according to current definitions [21]. The exclusion criteria were patients using antibiotics for more than 48 h at the time of screening, severe immunosuppression, patients under full and exclusive palliative care, death expectancy for the next 24 h, diagnose of infections known to require prolonged antibiotic therapy, and patients that underwent major surgery in the previous 5 days. For details of definitions and justifications of inclusion and exclusion criteria, see Additional file 1.

Eligible patients were randomly assigned to one of two groups: a CRP-guided therapy or a control group. Randomization was performed individually at an allocation rate of 1:1, using a computer-generated random number table that was sequenced in enumerated and sealed brown envelopes. The random allocation sequence was generated and supervised by a researcher not involved with the inclusion, follow-up, or analysis of the data. Patients were screened, randomized, and assigned to the groups by the principal investigator and assistants. Due to the nature of the intervention, the investigators and the assistant physicians were aware of the group in which the patients had been included. Preparation and conduction of the study followed the recommendations of the CONSORT Statement [22].

#### Intervention

The main purpose of this study was to evaluate if a strategy of CRP-guided therapy adds value to an already rational clinical practice of antibiotic use, applied to all study participants. Accordingly, in the control group, decisions about treatment time were taken according to the best evidence established in the literature for duration of antibiotic treatment [1–6]. The study protocol provided that antibiotic therapy should be discontinued in the control group when the patient reached clinical criteria for suspension, which did not include CRP levels and was based on clinical improvement, microbiological results, and stipulated time according to infectious focus. This stipulated duration is objectively described in Additional file 2. Apart from the values used in the diagnostic workup for infection, CRP levels were not provided to the clinical assistants and research teams during the antibiotic therapy period in this group of patients.

**Fig. 1:**
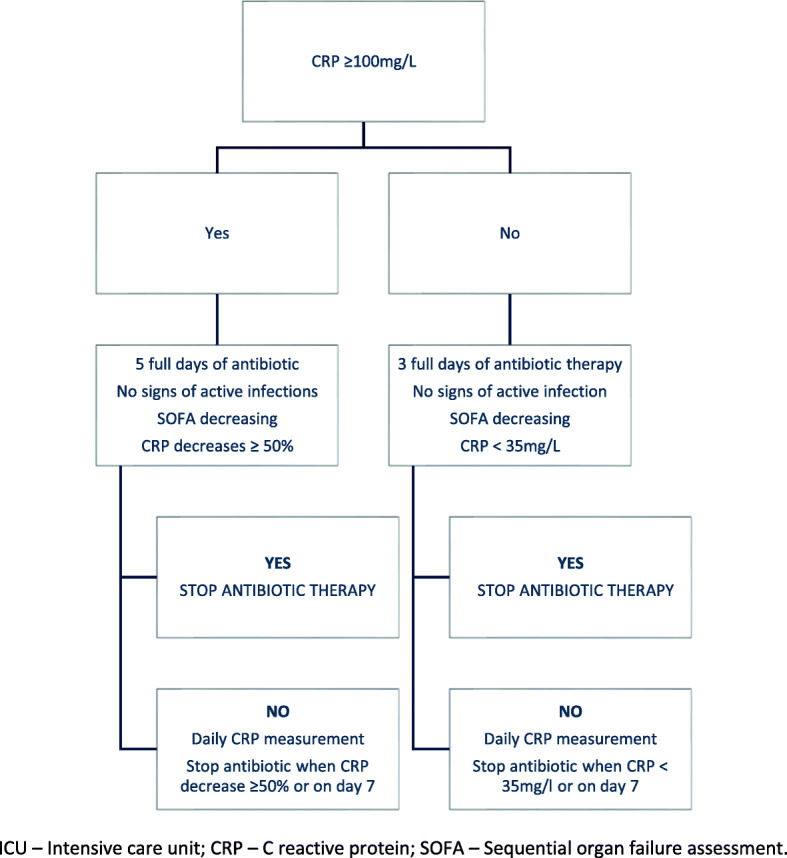
Decision-making flowchart for antibiotic discontinuation based on CRP levels. ICU, intensive care unit; CRP, C-reactive protein; SOFA, Sequential Organ Failure Assessment

In the intervention group, the duration of antibiotic therapy was defined through a clinical protocol based on the daily serum levels of CRP and patient’s clinical evolution (Fig. 1). The researchers recommended discontinuation of antibiotic treatment when the criteria in the study protocol were obtained. For patients with persistently elevated CRP serum levels but with clinical improvement and absence of signs of active infection, the duration of antibiotic therapy was the same as that suggested for the control group.

For both groups, the study protocol determined that patients with bacteremia received at least five full days of adequate antibiotic coverage. In all cases included, a strategy regarding the antibiotic therapy was recommended to the ICU teams. However, any decision on antimicrobial suspension was ultimately the responsibility of clinical assistants, who were allowed to keep or interrupt antibiotics at their discretion in both groups. We considered “non-adherence to the protocol” any case in where the treating team chose not to follow the investigators’ recommendations, either stopping antibiotics earlier or later than recommended by the protocol. The study protocol established longer antibiotic treatments independently of the levels of the biomarker (in the intervention group) or of the pre-determined time (in the control group) in cases of unfavorable clinical evolution, maintenance of uncontrolled focus, and clinical or microbiological findings that require extended antibiotic therapies. These cases were monitored daily by the research team, who recommended discontinuation of antibiotics as soon as possible. For both groups, a strategy of antibiotic de-escalation was recommended whenever possible.

### Outcomes and data collection

Patients were followed up by researchers from the time of inclusion until hospital discharge, death, or up to 90th day, whichever occurred first. Each patient was included only once.

The primary outcome analyzed was the duration of antibiotic therapy of the index infection episode. The sample size calculation was done with 5% alpha error and 80% power. A previous study comparing the duration of antibiotic therapy in septic patients under the guidance of CRP versus PCT revealed that the mean duration of treatment was 7.2 ± 3.5 days for the CRP group and 8.1 ± 3.7 days for the PCT group [19]. For the present study, the expected mean for the control group was estimated from the mean observed in the PCT group in the cited study (~ 8 days), and the expected days of treatment in the CRP group was reduced to 6 days, keeping the standard deviations (SDs) found and obtaining an effect size of 0.55 by *t* test for independent samples. Thus, we estimated the need for 53 patients per group, plus a 15% correction due to a non-normal distribution of data, totaling the sample size as 122 patients. Detailed calculation is described in Additional file 3.

Secondary outcomes were mortality for any cause on intensive care and at the 28th day, frequency of therapeutic failure and recurrence of infections, frequency of sequential nosocomial infections, the occurrence of sequential infections by multi-resistant bacteria, time in mechanical ventilation, and length of in hospital and intensive care stay. The definitions adopted for the response variables are described in Additional file 4.

The registry of the laboratory and clinical information was carried out prospectively. The data included demographic information; the scores Charlson [23], Sequential Organ Failure Assessment (SOFA) [21], Simplified Acute Physiology Score 3 (SAPS 3) [24], and Acute Physiology and Chronic Health Evaluation II (APACHE II) [25]; sepsis classification [21]; infections types and microbiology [26]; and choice of antimicrobials and respective length of use. CRP was measured in the serum at the inclusion date and daily within the first 7 days of follow-up, using the test for quantitative determination of serum CRP concentration (Vitros-Johnson & Johnson, USA).

### Statistical analyses

All analyses were performed using R version 3.1.1 (R Foundation for statistical computing, Vienna, Austria) and SPSS (SPSS Statistics 20.0, Armonk, NY: IBM Corp. USA). Categorical variables are presented according to their absolute and relative frequencies. Continuous variables (non-normal distribution) are presented as median and interquartile range 25–75% (Q1–Q3).

Primary and secondary outcomes were primarily analyzed according to intention to treat (ITT). Both groups were compared using chi-square or Student’s *t* test/Mann-Whitney *U* test as indicated. For additional analysis of the primary outcomes, a cumulative curve of antibiotic discontinuation was compared between both groups (time-to-event analysis) using the Wilcoxon test [27]. When relevant, the results are presented by odds ratio and 95% confidence intervals. Two-tailed tests and a significance level of 0.05 were used for all analyses.

## Results

### Baseline demographics and clinical characteristics

**Fig. 2:**
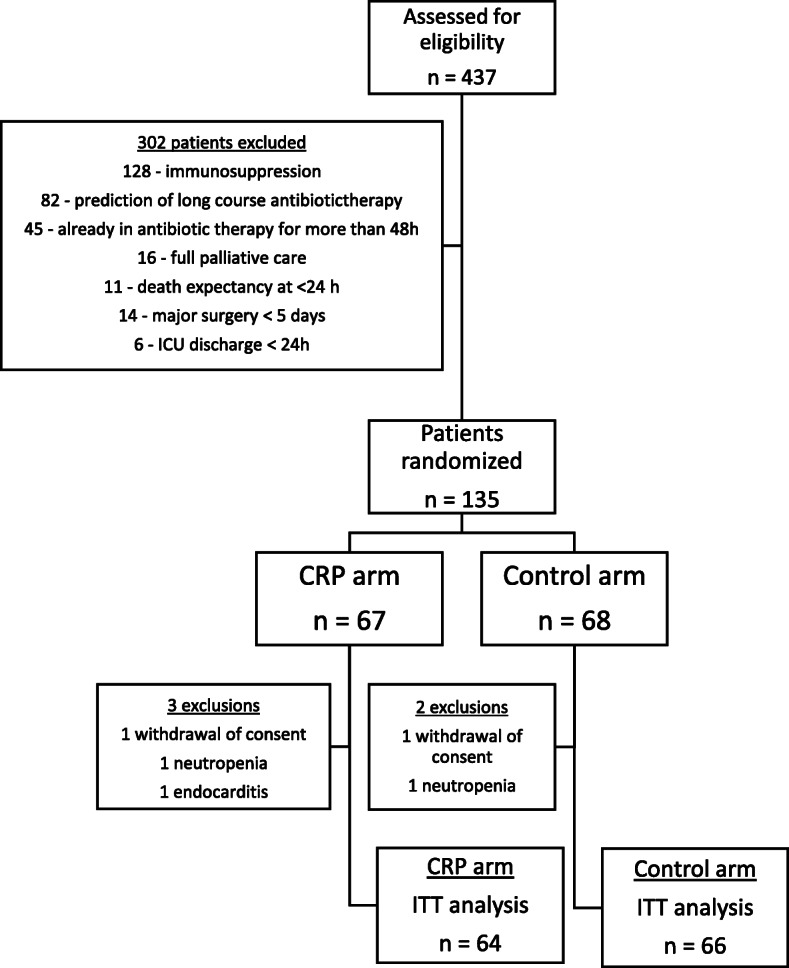
Inclusion flow diagram

We evaluated 437 patients for eligibility, and a total of 135 patients were randomized. After randomization, five patients were excluded, and 130 patients were included in the intention-to-treat analysis (Fig. 2).

Demographics and clinical characteristics at inclusion were similar between patients of the two groups (Table 1). The established empiric antimicrobial therapy was adequate in 90% of the cases [28]. The values of CRP upon admission and during the first 10 days of follow-up did not reveal significant differences between the groups (Additional file 5).

**Table 1:** Baseline demographic and clinical characteristics of the population

Characteristics	Overall (*n* = 130)	CRP group (*n* = 64)	Control (*n* = 66)	*p* value
**Age, years (median, Q1–Q3)**	61 (51–68)	62 (53–68)	60 (49–70)	0.513
**Age, years (mean, SD)**	58.6 (± 15.8)	60.2 (± 14)	57 (± 17.3)	0.252
**Female sex,*n*(%)**	62 (47.7%)	30 (46.9%)	32 (48.5%)	0.854
**Medical patient,*n*(%)**	107 (82.3%)	53 (82.8%)	54 (81.8%)	0.882
**Comorbidities,*n*(%)**				
Heart failure	26 (20%)	14 (21.9%)	12 (18.2%)	0.599
Solid malignancy	15 (11.5%)	7 (10.9%)	8 (12.1%)	0.934
Hematologic malignancy	2 (1.5%)	1 (1.6%)	1 (1.5%)	1.0
COPD	13 (10%)	8 (12.5%)	5 (7.6%)	0.348
Cirrhosis	14 (10.8%)	8 (12.5%)	6 (9.1%)	0.531
Chronic renal failure	20 (15.4%)	11 (17.2%)	9 (13.6%)	0.575
Dialytic chronic renal failure	8 (6.2%)	5 (7.8%)	3 (4.5%)	0.438
Hypertension	69 (53.1%)	38 (59.4%)	31 (47%)	0.157
Diabetes	44 (33.8%)	25 (39.1%)	19 (28.8%)	0.216
PLWHA	1 (0.8%)	1 (1.6%)	0 (0%)	0.308
**Charlson (median, Q1–Q3)**	4 (2–5)	4 (2–5)	3 (1.8–6)	0.126
**SAPS 3 (median, Q1–Q3)**	59 (50–70)	62 (49–70)	59 (51–70)	0.119
**APACHE II (median, Q1–Q3)**	18 (13–22)	18 (14–23)	16 (13–21)	0.909
**SOFA (median, Q1–Q3)**	7 (5–9)	7 (4–9)	6 (5–9)	0.356
Respiration	2 (1–2)	2 (1–2)	2 (1–2)	0.618
Coagulation	0 (0–1)	0 (0–2)	0 (0–1)	0.175
Liver	0 (0–1)	0 (0–1)	0 (0–1)	0.584
Cardiovascular	1 (0–4)	1 (0–4)	2 (0–4)	0.458
CNS	1 (0–2)	1 (0–2)	0 (0–1)	0.071
Renal	1 (0–3)	1 (0–4)	1 (0–3)	0.678
**Sepsis classification,*n*(%)**				0.502
Infection	8 (6.2%)	5 (7.8%)	3 (4.5%)	
Sepsis	80 (61.5%)	41 (64.1%)	39 (59.1%)	
Septic shock	42 (32.3%)	18 (28.1%)	24 (36.4%)	
**First infection episode,*n*(%)**	115 (88.5%)	54 (84.4%)	61 (92.4%)	0.151
**Site of infection,*n*(%)**				0.228
Lungs	58 (44.6%)	27 (42.2%)	31 (47%)	
Abdomen	29 (22.3%)	13 (20.3%)	16 (24.2%)	
Urine	20 (15.4%)	8 (12.5%)	12 (18.2%)	
Catheter	6 (4.6%)	3 (4.7%)	3 (4.5%)	
Skin and soft tissue	5 (3.8%)	5 (7.8%)	0 (0%)	
CNS	2 (1.5%)	2 (3.1%)	0 (0%)	
Others	10 (7.7%)	6 (9.4%)	4 (6%)	
**Nosocomial infection,*n*(%)**	74 (57%)	39 (61%)	35 (53%)	0.363
**Positive microbiology,*n*(%)**	66 (50.8%)	29 (45.3%)	37 (56.1%)	0.220
**Positive blood culture,*n*(%)**	40 (30.8%)	16 (25%)	24 (36.4%)	0.160
**MDR infections,*n*(%)**	29 (22.3%)	17 (26.5%)	12 (18.2%)	0.251
**Appropriate empirical therapy,*n*(%)**	117 (90%)	58 (90.6%)	59 (89.4%)	0.815
**Ventilatory support first 72 h,*n*(%)**	77 (59.2%)	39 (60.9%)	38 (57.6%)	0.697
**Hemodialysis first 72 h,*n*(%)**	33 (25.4%)	18 (28.1%)	15 (22.7%)	0.480
**Inotropes first 72 h,*n*(%)**	16 (12.3%)	9 (14.1%)	7 (10.6%)	0.549
**Steroids first 72 h,*n*(%)**	27 (20.8%)	16 (25%)	11 (16.7%)	0.242
**Steroids for septic shock,*n*(%)**	4 (3%)	2 (3.1%)	2 (3%)	0.369
**Vasopressor first 72 h,*n*(%)**	62 (47.7%)	31 (48.4%)	31 (47%)	0.867
**Lactate mg/dl (median, Q1–Q3)**	2 (1.5–2.8)	1.9 (1.4–2.4)	2 (1.6–3)	0.599
**Leucocytes g/dl × 10**^**3**^**(median, Q1–Q3)**	12 (8.7–16.8)	11.7 (8–15.1)	12.6 (9–17.8)	0.180
**Neutrophil g/dl × 10**^**3**^**(median, Q1–Q3)**	8.7 (6–13.3)	8.7 (5.3–12.3)	8.8 (6–14.4)	0.591
**CRP mg/L (median, Q1–Q3)**	227 (137–334	199 (75–313)	234 (151–332)	0.095

*COPD* chronic obstructive pulmonary disease, *PLWAH* people living with HIV and AIDS, *SAPS-3* Simplified Acute Physiology Score 3, APACHE II Acute Physiology and Chronic Health Disease Classification System II, *SOFA* Sequential Sepsis-Related Organ Failure Assessment, *CNS* central nervous System, *CRP* C-reactive protein

### Outcome results: intention-to-treat analysis

**Fig. 3:**
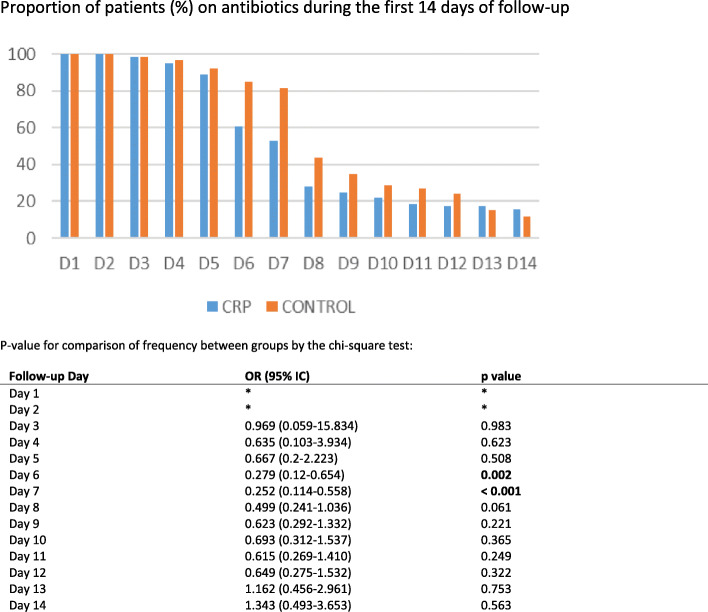
Proportion of patients (%) on antibiotics during the first 14 days of follow-up. *p* value for comparison of frequency between groups by the chi-square test

The ITT analysis revealed that the median duration of antibiotic therapy in the index episode of infection was similar in both groups, with first and third quartiles showing higher values in the control group: 7 (5–8.8) days in the CRP group versus 7 (7–11.3) days in the control group (*p* = 0.011) (Table 2; Additional file 6). In the CRP group, more patients had their antimicrobial therapy suspended up to the fifth day of follow-up compared to the control (35.9% vs. 10.6%, OR 4.7, 95% CI 1.9–12, *p* = 0.001). The proportion of patients taking antibiotic therapy over the first 14 days of follow-up is presented in Fig. 3.

**Table 2:** Primary and secondary endpoints by treatment arm in the intention-to-treat analysis

Outcomes	Overall (*n* = 130)	CRP group (*n* = 64)	Control (*n* = 66)	*p* value
Primary outcomes				
Duration of antibiotic therapy (median, Q1–Q3)	7 (5–10)	7 (5–8.8)	7 (7–11.3)	0.011
Duration of antibiotic therapy (mean, ± SD)	9 (± 8)	8 (± 6.3)	10 (± 9.3)	0.264
Secondary outcomes				
Total exposure to antibiotic, days (median, Q1–Q3)	8 (7–17)	8 (6–19)	8.5 (7–16)	0.564
Antibiotic-free period, days/100 live days (median, Q1–Q3)	47.5 (15.1–63.1)	51.6 (12.9–67.2)	40.6 (18.8–59.3)	0.252
De-escalation rate (%)	40 (30.7%)	19 (29.7%)	21 (31.8)	0.850
Length of stay in ICU, days (median, Q1–Q3)	8 (4–15)	8 (4–15)	8 (4–17)	0.414
Length of stay in hospital, days (median, Q1–Q3)	29 (15–47)	31.5 (16–53)	25.5 (15–43)	0.356
Length of mechanical ventilation support, days (median, Q1–Q3)	2.5 (0–9)	2 (0–9)	3 (0–9)	0.676
28th-day mortality, *n* (%)	33 (25.4%)	18 (28.1%)	15 (22.7%)	0.480
ICU mortality, *n* (%)	24 (18.5%)	12 (18.8%)	12 (18.2%)	0.933
Sepsis-related death, *n* (%)	25 (19.2%)	15 (23.4%)	10 (15.2%)	0.363
Recurrence of first infection, *n* (%)	4 (3.1%)	3 (4.7%)	1 (1.5%)	0.295
Sequential nosocomial infection, *n* (%)	43 (33.1%)	21 (32.8%)	22 (33.3%)	0.950
MDR pathogen infection, *n* (%)	18 (13.8%)	9 (14.1%)	9 (13.6%)	0.572

*ICU* intensive care unit, *MDR* multi-drug resistant

There was no between-group significant difference regarding the total days of antibiotic exposure during follow-up and antibiotic-free days. Similar results were found for ICU and hospital length of stay, mechanical ventilation time, intensive care and 28th-day mortality, sepsis-related death, recurrence of infection, new nosocomial infections, and infection with multidrug-resistant bacteria (Table 2).

**Fig. 4:**
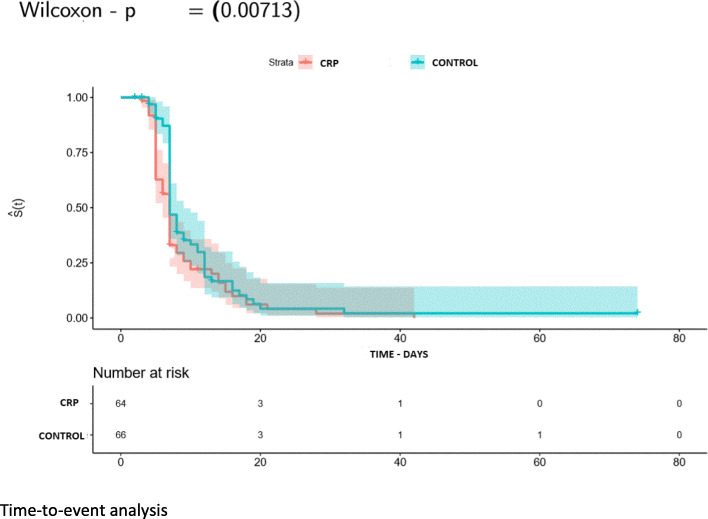
Cumulative curve of antibiotic suspension. Time-to-event analysis

In a time-to-event data analysis, with the target event defined as “antibiotic suspension” and censoring patients who did not experience this outcome (due to death or hospital discharge in use of antibiotic therapy), a significant lower antibiotic exposure for the index infection episode was observed in the CRP group (*p* = 0.007) (Fig. 4).

The workflow of interventions in each subgroup completed with outcome related to each of the subpopulations is depicted in Additional file 7.

### Outcome results: per-protocol and subgroup analysis

The rate of adherence to the protocol was 91% (92.2% CRP vs. 89.4% control, *p* = 0.764). Analysis per protocol revealed a reduction of 1 day in the median duration of antibiotic therapy for the index infection episode in the CRP group in comparison to the control (6 (5–8) days vs. 7 (7–10) days; *p* = 0.011) (Additional file 8). Similar results were observed in post hoc analysis restricted to patients with SAPS 3 less than or equal to 59 (*n* = 66) at admission, patients with community-acquired infections (*n* = 56), patients with lower-respiratory tract infections (*n* = 58), and those who had adequate initial empirical antimicrobial therapy (*n* = 117) (Additional file 8).

## Discussion

In this randomized clinical trial, we investigated the usefulness of a CRP-based protocol to reduce the duration of antibiotic therapy in critically ill patients undergoing an evidence-based judicious use of antibiotics strategy. We found lower antibiotic exposure in the intervention patients in comparison to controls, who were treated according to the best practice in antibiotic therapy [1–3, 5, 8], only when considering the index infection episode. For this first treated episode, despite a similar median time of therapy, there was a narrower distribution of this parameter in the CRP arm patients. Moreover, in the CRP group, more patients had their antimicrobial therapy suspended up to the fifth day of follow-up, with a significant lower antibiotic exposure in the time-to-event analysis. Finally, the analysis per protocol revealed a reduction of 1 day in the median duration of antibiotic therapy in the intervention group. It should be stressed that these findings did not translate into more antibiotic-free days or in a reduced antimicrobial exposure.

Given the benefits offered by the rational use of antibiotics, including the reduction of multiresistant bacteria [5, 29, 30], treatment costs [31], frequency of adverse effects [32], and less interference with microbiome, objective criteria to define the ideal treatment length is warranted. CRP is a low-cost and affordable biomarker [10], routinely used in intensive care, that has been shown relate to prognosis in studies involving different populations with serious infectious conditions [17, 20, 33, 34].

Previous studies using biomarkers, notably PCT, included control groups in which the therapeutic strategy was freely determined by the assistant team. This strategy may have led to excessively long treatment duration of the control groups [10], which varied from 10 [11] to 15 days [12]. A meta-analysis involving data from more than 4000 patients on PCT-guided antibiotic therapy of acute respiratory infections revealed that the PCT-guided group was treated for 7 days in comparison with the control, which received 10 days treatment, or 14 days for patients in intensive care settings [35]. In our study, we used the best standard of care in the control group, not the usual care as described above.

More recently, two studies have tested the usefulness of biomarker-guided antibiotic therapy compared to controls using shorter therapies. In a single-center study, Oliveira et al. found that a PCT-based protocol was not superior to a protocol based on serum CRP levels for reducing the use of antibiotics in sepsis. It is worth highlighting the fact that in this study, the researchers originally stipulated a maximum of 7 days for the duration of the therapy, independently of the levels of biomarkers [19]. In a larger Dutch study, de Jong et al. showed the usefulness of PCT to reduce the duration of antibiotic therapy in critically ill patients, with 5 days as the median treatment time compared to 7 days for controls [14]. It is noteworthy that the population included in the present work was significantly more severely ill than the patients included in the Dutch study [14] (septic shock 32.3% vs. 18.5%, respectively), which reinforces the value of our results, even if incipient.

Although our study showed no difference in median duration of antibiotics in the ITT analysis, there were more patients which received shorter durations of antibiotics in the CRP arm. Also, there was less exposure in the CRP group in the cumulative curve of antibiotic suspension for the first infection episode. Further, 1-day reduction in median duration of antibiotic treatment was found in the per-protocol analysis and in different post hoc analyses of subgroups. Specifically, in patients of lower severity and complexity (e.g., community-acquired infections and SAPS-3 < 50%), the difference found may be justified by the easier application of the decision flowchart. In patients with respiratory tract infection, there is an already known better CRP performance in patients with pneumonia [36]. Patients who had appropriate initial empirical antibiotic therapy may have presented better results by the lower interference of inadequate initial antibiotics in treatment time [28, 37]. These preliminary findings reinforce the potential role of a CRP-guided protocol in reducing antibiotic exposure in hospitalized infected patients. Interestingly, in a recent published meta-analysis, authors found that the use of PCT algorithms to guide antibiotic therapy was associated with increased survival especially when combined with a CRP-guided strategy [16].

The rate of adherence to the protocol reported herein was higher than that reported in previous clinical trials [13, 14]. Patients were included when in intensive care and followed up until hospital discharge or death. Therefore, interventions were also applied in other hospital units. This strategy allowed the high rates of adhesion to the protocol and proved feasible from the logistic point of view.

Despite such promising findings, other relevant investigated outcomes such as antibiotic-free days and total time of antibiotic therapy during follow-up revealed similar between groups. Also, no statistically significant differences were found in safety and survival outcomes. These findings suggest that CRP-guided therapy may be effective and safe in some specific scenarios, although further studies, with a sample size powered for safety analysis, should be conducted to confirm this hypothesis. Ideally, in settings with a less complex patient profile, where single courses of antibiotics are held more often.

Our study has several limitations that should be mentioned. This was a single-center study, restricted to two intensive care units of a high complexity hospital. Therefore, the findings lack external validity and cannot be extrapolated to other populations. In addition, the inherent open design may have biased the results, favoring the alternative hypothesis. Third, there was a high rate of non-inclusion among the patients evaluated for potential eligibility. Although this scenario has been observed in several similar studies [11, 14], this fact limits the population to which the protocol can be applied, especially immunosuppressed population. It remains unclear how these patients, including those with immunosuppressive dose corticosteroid therapy, respond to CRP-guided antibiotic therapy. Also, although infection with non-fermenting Gram-negative bacteria was not an exclusion criterion, patients with this kind of infection were poorly represented in this study. Fourth, there was an apparent trend towards higher mortality in the CRP-guided therapy group, with no statistically significant difference. However, sepsis-associated mortality was quite similar between the two groups, as well as recurrent infection rates. Also, mortality rate of patients who had early suspension of antibiotics, according to study protocols, was lower than the overall mortality rate and similar between intervention and control groups (Additional file 7).

## Conclusions

A protocol based on daily monitoring of CRP levels may support a tailored time of antibiotic therapy in critically ill patients in a single infection episode, but without reducing the total exposure of these patients to antimicrobials. The subtle time reduction observed in the group of patients undergoing CRP-guided therapy may be potentially impacting in less complex scenarios, since it was observed even in a scenario of judicious use of antibiotics. CRP-guided strategy is feasible. Further studies are needed to add up to these findings, to properly assess safety outcomes and to evaluate the real impact of this strategy in clinical practice.

## Supplementary information

**Additional file 1.** Inclusion and exclusion criteria.

**Additional file 2.** Antibiotic therapy time provided for the control (best practice) group.

**Additional file 3.** Sample size calculation.

**Additional file 4.** Definitions of the response variables.

**Additional file 5.** Serial measurements of C-reactive protein concentrations in both study groups.

**Additional file 6.** Days of antibiotic therapy in the index infection episode according to the inclusion group - Intention-to-treat analysis.

**Additional file 7.** Workflow of interventions and outcomes.

**Additional file 8.** Duration of antibiotic therapy for the index infectious episode: analysis per protocol and other subgroups.

## Data Availability

The datasets used and analyzed during the current study are available from the corresponding author on reasonable request.

## Abbreviations

APACHE II: Acute Physiology and Chronic Health Evaluation II
CAP: Community-acquired pneumonia
CI: Confidence interval
CRP: C-reactive protein
ICU: Intensive care unit
ITT: Intention to treat
OR: Odds ratio
PCT: Procalcitonin
Q1-Q3: Interquartile range
SAPS 3: Simplified Acute Physiology Score 3
SOFA: Sequential Organ Failure Assessment
UFMG: *Universidade Federal de Minas Gerais*
VAP: Ventilator-associated pneumonia
