# Supplementary material for: Duration of antibiotic therapy in critically ill patients: a randomized controlled trial of a clinical and C-reactive protein-based protocol versus an evidence-based best practice strategy without biomarkers
Source: Crit Care. 2020 Jun 1;24:281. doi: 10.1186/s13054-020-02946-y (PMC7266125; doi:10.1186/s13054-020-02946-y)
Supplement: Supplementary file 1 — Additional file 1. Inclusion and exclusion criteria. [file 13054_2020_2946_MOESM1_ESM.docx]

**Additional file 1**

Inclusion criteria

- Age greater than or equal to 18 years;

- Being hospitalized in one of the intensive care units participating in the study, with a prospect of staying longer than 24 hours;

- Signature of informed consent, which could be done by a family member or carer, if the patient was not aware or with adequate conditions of discernment;

- Patient with clinical suspicion or with microbiological confirmation of infection, sepsis or septic shock;

Infection was defined by the clear expression of the suspicion of an infectious condition by the physicians, associated with the initiation of antimicrobial treatment and following a directed propaedeutic;

Sepsis was defined by the presence of at least one new organ dysfunction associated with infection, characterized by the variation of at least two points in the SOFA (Sequential [Sepsis-related] Organ Failure Assessment);

Septic shock was defined as persistent hypotension requiring vasopressors for maintenance of mean arterial pressure (MAP) greater than 65 mmHg associated with a lactate level greater than 2 mmol / L despite adequate volume resuscitation;

The microbiological confirmation of the infectious condition was defined by the isolation of at least one microorganism considered to be pathogenic, no more than 48 hours before inclusion in the study, and to which the physicians directed an antimicrobial therapy.

Exclusion Criteria

- Use of antibiotics supposedly or proven to be effective against the infectious process in question for more than 48 hours at the time of screening;

- Severely immunosuppressed patients (HIV infection with CD4 + lymphocytes <200 / mm3, severe neutropenia <500 / mL, post-transplantation of solid organ or bone marrow in immunosuppressive therapy, undergoing myelosuppressive chemotherapy in the last 28 days, patients with inflammatory diseases or autoimmune with chronic use of immunosuppressants, including **previous** use of corticosteroids - prednisone dose or equivalent greater than 10mg / day for ≥ 30 days or 40mg / day for ≥ 10 days - or in pulse therapy). **Patients receiving corticosteroids for the treatment of septic shock were not excluded.**

- Patients under full and exclusive palliative care;

- Patients with death expectancy for the next 24 hours;

- Patients who at the time of screening present a diagnosis of infections that would be known to require prolonged antibiotic therapy, such as bacteremia by *Staphylococcus aureus* or *Candida* spp; confirmed or strongly suspected bacterial endocarditis; chronic osteomyelitis; abscesses not drained;

- Patient with polytrauma in the last five days, due to changes in biomarker kinetics in the period related to trauma insult;

- Patient undergoing major surgery five days previous to the inclusion, due to changes in biomarker kinetics in the postoperative period related to surgical insult, except in cases of surgeries performed to control the infectious focus in question (eg exploratory laparotomy in patients with acute inflammatory abdomen).
