# Supplementary material for: Duration of antibiotic therapy in critically ill patients: a randomized controlled trial of a clinical and C-reactive protein-based protocol versus an evidence-based best practice strategy without biomarkers
Source: Crit Care. 2020 Jun 1;24:281. doi: 10.1186/s13054-020-02946-y (PMC7266125; doi:10.1186/s13054-020-02946-y)
Supplement: Supplementary file 2 — Additional file 2. Antibiotic therapy time provided for the control (best practice) group. [file 13054_2020_2946_MOESM2_ESM.docx]

**Additional file 2**

Antibiotic therapy time provided for the control (best practice) group

| **Infectious type or focus** | **Estimated time of Antibiotic Therapy** |
| --- | --- |
| Community-acquired pneumonia | 7 days |
| Non-VAP hospital pneumonia | 7 days |
| VAP Pneumonia hospital | 7 days |
| Pyelonephritis | 7 days |
| Intra-abdominal infection - drained focus | 5 days |
| Bacteremia | 7 days |
| Non-fermenting MO pneumonia | 10 days |
| Pneumonia by KPC | 10 days |
| Necrotizing pneumonia ** | 14 days |

VAP - mechanical ventilation associated pneumonia; MO - microorganism; KPC - gram negative bacteria producing carbapenemase. * Confirmed or highly suspected etiology due to prior colonization. ** Confirmed by chest computed tomography.
