# Supplementary material for: Duration of antibiotic therapy in critically ill patients: a randomized controlled trial of a clinical and C-reactive protein-based protocol versus an evidence-based best practice strategy without biomarkers
Source: Crit Care. 2020 Jun 1;24:281. doi: 10.1186/s13054-020-02946-y (PMC7266125; doi:10.1186/s13054-020-02946-y)
Supplement: Supplementary file 3 — Additional file 3. Sample size calculation. [file 13054_2020_2946_MOESM3_ESM.docx]

**Additional file 3**

Sample size calculation

A previous study comparing the duration of antibiotic therapy in septic patients under the guidance of CRP versus PCT revealed that the mean duration of treatment was 7.2 ± 3.5 days for the CRP group and 8.1 ± 3.7 days for the PCT group^19^. For the present study, the expected mean for the control group was estimated from the mean observed in the PCT group in the cited study (~ 8 days), and the expected days of treatment in the CRP group was reduced to 6 days, keeping the standard deviations (SDs) found and obtaining an effect size of 0.55 by t-test for independent samples. Thus, we estimated the need for 53 patients per group. Parametric tests were used for sample calculation. However, non-normal distribution of variables and the need to use nonparametric tests for the main statistical analyzes were expected. For this reason, we added a 15% correction totaling the sample size as 122 patients*. This calculation was done with 5% alpha error and 80% power.

* Method described in: Erich L. Lehmann, Nonparametrics: Statistical Methods Based on Ranks, Revised, 1998, ISBN = 9780139977350, pages 76-81.
