# Supplementary material for: Duration of antibiotic therapy in critically ill patients: a randomized controlled trial of a clinical and C-reactive protein-based protocol versus an evidence-based best practice strategy without biomarkers
Source: Crit Care. 2020 Jun 1;24:281. doi: 10.1186/s13054-020-02946-y (PMC7266125; doi:10.1186/s13054-020-02946-y)
Supplement: Supplementary file 4 — Additional file 4. Definitions of the response variables. [file 13054_2020_2946_MOESM4_ESM.docx]

**Additional file 4 - Definitions of the response variables**

The definitions adopted for the response variables were:

Antibiotic use duration was assessed as follows: (1) duration of antimicrobial therapy of the episode index, measured as days of the therapeutic course for the focus that led to inclusion in the study; (2) total days of exposure to antibiotics considering all therapeutic courses during follow-up; (3) antibiotic free days during the follow-up, corrected for 100 days (nº of days of follow-up without antibiotics/n° of total days of follow-up – time of inclusion until hospital discharge. This proportion was corrected to a denominator of 100, in a simple rule of three). An antibiotic day exposure was defined as the period of continuous administration of a single drug, with all doses administered at the expected intervals, without interruption for at least 24 hours. A day without antibiotics was defined as the period of at least 24 hours without the administration of antibiotics for a given patient (Oliveira et al., 2013; De Jong et al., 2016). In this study, a therapeutic course of antibiotics consisted of the administration of one or more antibiotics, being of the same or of different classes, but indicated for the same clinical reason, and without interruption for more than 24 hours. Topical administration of antibiotics was not considered in this analysis.

- Rate of therapeutic failure with recurrence of infection (in N recurrent infections per 100 patients). In this study we defined therapeutic failure and recurrence of infection as the persistence or recurrence of signs and symptoms of infection that motivated the resumption of the therapeutic course of antibiotics with less than 48 hours after the initial suspension of the same focus.

- Survival or death were evaluated during intensive care hospitalization and at 28 days of follow-up. Deaths were classified as related or not to sepsis.

- Time of mechanical ventilation, total follow-up time, and intensive care (full days).

- Rate of nosocomial infection (in N of nosocomial infections per 100 patients). Nosocomial infection is defined as infection acquired after the patient's hospitalization, and any clinical manifestation of infection is present after 48 hours after admission, when there is no clinical and / or laboratory evidence of infection at the time of admission (Garner et al., 1988). We documented the nosocomial infections that occurred after inclusion of the patient in the study and the beginning of follow-up of the patient during the follow-up.

- Infection with multiresistant bacteria (in clinical isolates per 100 patients). Multiresistance is defined as any organism that is resistant to at least three classes of antibiotics, without considering the classes to which it would be intrinsically resistant (e.g. MRSA) (Magiorakos et al., 2012). Infection by the multiresistant microorganism is defined by the isolation of the same accompanied by clinical criteria infectious that motivate the direction of the antimicrobial therapy.

- Sepsis-associated mortality: defined by the cause-of-death record made by the clinical assistants.

- The definitions of appropriateness of empiric antibiotics ware based on the reference number 28 (Kumar A et al., Chest. 2009). The main criteria were:

“Rules to Assign Clinical Significance to Microbial Isolates:

1. Clinically significant isolates from either local site and/orblood cultures were required to have been obtained within 48 h of the onset of shock.

2. The following were considered to represent clinically significant isolates:

a. A blood culture positive for any pathogen other than coagulase-negative staphylococci or other skin contaminants;

b. Any growth from a normally sterile site (eg, gall bladder, bronchial lavage, peritoneal, pleural fluid, or operative tissue specimen) apart from coagulase-negative staphylococci and other skin contaminants;

c. Growth of a pathogen in a sputum sample from a patient with respiratory signs and symptoms, or a new infiltrate seen on chest radiography, with no other likely source of infection;

d. Growth of a pathogen in a urine sample with either local clinical symptoms or in the absence of a more plausible clinical infection site;

e. Growth from a deep biopsy specimen or a deep aspirate of a finding in soft tissue or skin;

3. Candida lung isolates were considered to be colonizers unless also isolated from multiple other normally sterile sites in which case disseminated infection was diagnosed.

4. Staphylococcus epidermidis was uniformly considered to be incapable of causing septic shock. Other coagulase-negative staphylococci were similarly considered to be unlikely to cause septic shock unless present as a sole isolate in multiple blood cultures in the absence of evidence of endovascular infection.

The use of appropriate antimicrobial therapy with in vitro activity appropriate to isolated pathogenic organisms was determined.

If a pathogenic organism was not isolated, the use of appropriate antimicrobial therapy for the underlying clinical syndrome was determined. For culture-negative infection episodes, appropriate therapy was deemed to be initiated when antimicrobial agents consistent with broadly accepted norms for empiric management of the typical pathogens for the clinical syndrome (in the context of host immune/health status, environmental factors, and local flora) were administered.”
