# Supplementary material for: Duration of antibiotic therapy in critically ill patients: a randomized controlled trial of a clinical and C-reactive protein-based protocol versus an evidence-based best practice strategy without biomarkers
Source: Crit Care. 2020 Jun 1;24:281. doi: 10.1186/s13054-020-02946-y (PMC7266125; doi:10.1186/s13054-020-02946-y)
Supplement: Supplementary file 5 — Additional file 5. Serial measurements of C-reactive protein concentrations in both study groups. [file 13054_2020_2946_MOESM5_ESM.docx]

**Additional file 5**

Serial measurements of C-reactive protein concentrations in both study groups

**
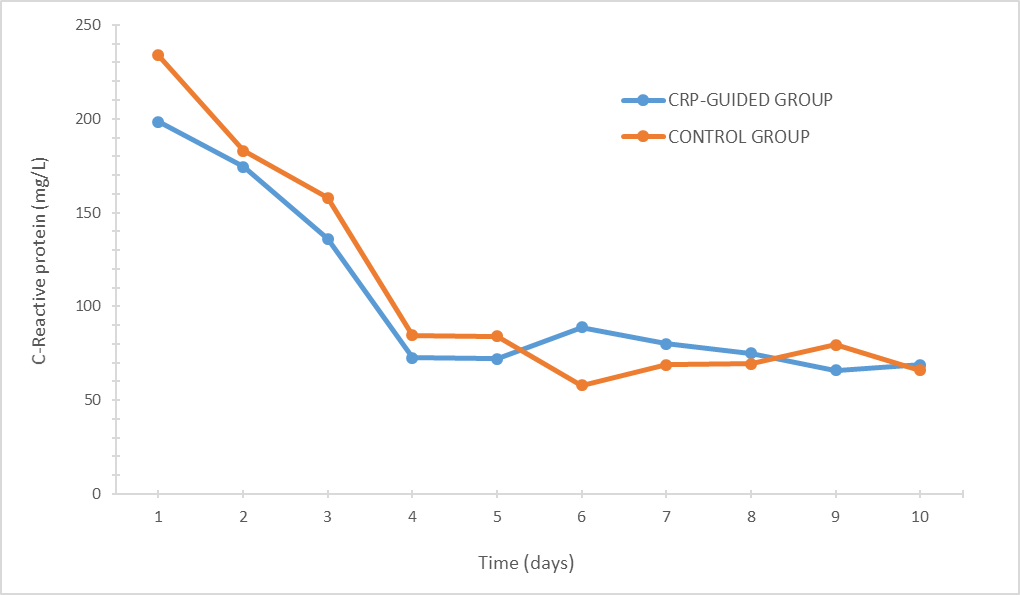
**
