# Supplementary material for: Duration of antibiotic therapy in critically ill patients: a randomized controlled trial of a clinical and C-reactive protein-based protocol versus an evidence-based best practice strategy without biomarkers
Source: Crit Care. 2020 Jun 1;24:281. doi: 10.1186/s13054-020-02946-y (PMC7266125; doi:10.1186/s13054-020-02946-y)
Supplement: Supplementary file 6 — Additional file 6. Days of antibiotic therapy in the index infection episode according to the inclusion group - Intention-to-treat analysis. [file 13054_2020_2946_MOESM6_ESM.docx]

**Additional file 6**

Days of antibiotic therapy in the index infection episode according to the inclusion group - Intention-to-treat analysis

**
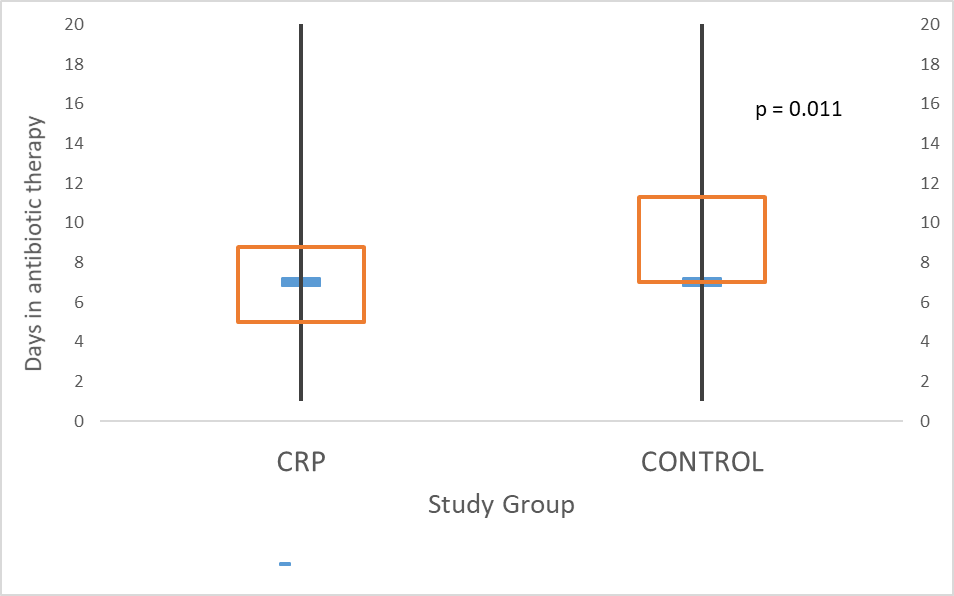
**

Box plots with median days of treatment, interquartile ranges and 10 and 90 percentiles according to inclusion group.
