# Supplementary figures and images for: Duration of antibiotic therapy in critically ill patients: a randomized controlled trial of a clinical and C-reactive protein-based protocol versus an evidence-based best practice strategy without biomarkers
Source: Crit Care. 2020 Jun 1;24:281. doi: 10.1186/s13054-020-02946-y (PMC7266125; doi:10.1186/s13054-020-02946-y)

**Additional file 7**

Workflow of interventions and outcomes


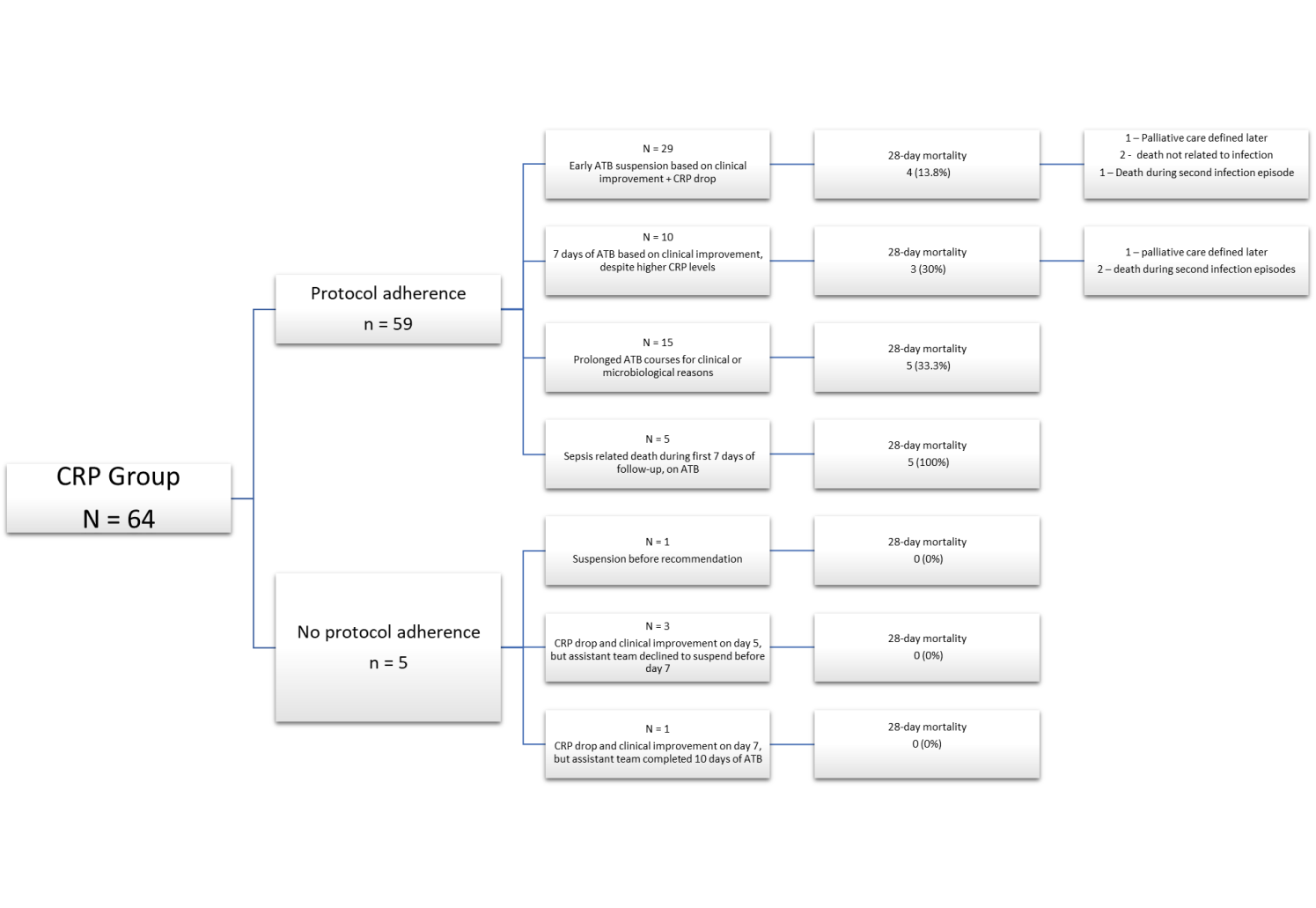


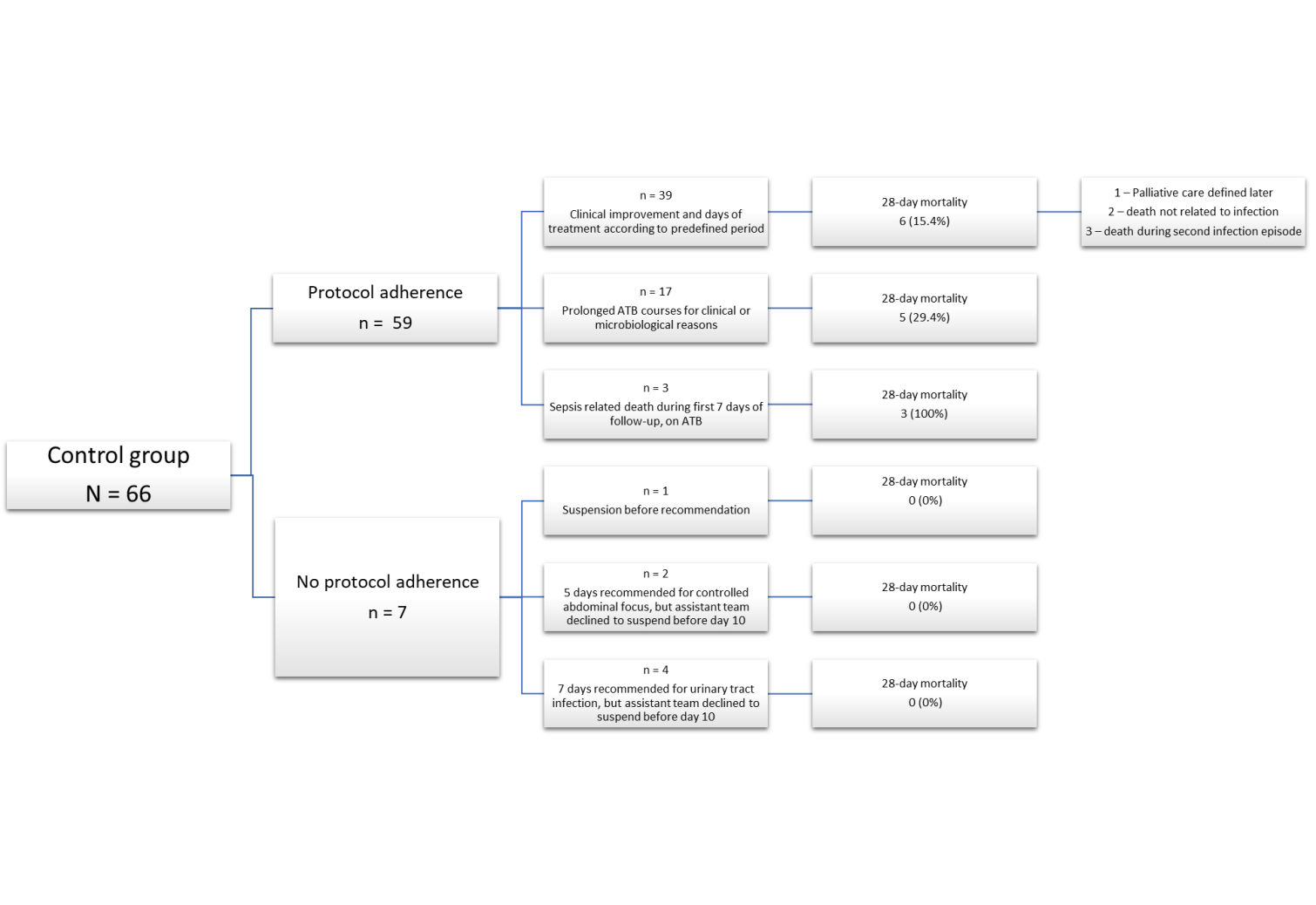

Supplement: Supplementary file 7 — Additional file 7. Workflow of interventions and outcomes. [file 13054_2020_2946_MOESM7_ESM.docx]
