# Supplementary material for: Duration of antibiotic therapy in critically ill patients: a randomized controlled trial of a clinical and C-reactive protein-based protocol versus an evidence-based best practice strategy without biomarkers
Source: Crit Care. 2020 Jun 1;24:281. doi: 10.1186/s13054-020-02946-y (PMC7266125; doi:10.1186/s13054-020-02946-y)
Supplement: Supplementary file 8 — Additional file 8. Duration of antibiotic therapy for the index infectious episode: analysis per protocol and other subgroups. [file 13054_2020_2946_MOESM8_ESM.docx]

**Additional file 8 -** Duration of antibiotic therapy for the index infectious episode: analysis per protocol and other subgroups

| Subgroups | Overall  (N)  (ATB days, mediam, Q1-Q3)  (ATB days, | CRP Group  (N)  (ATB days, median, Q1-Q3) | Control  (N)  (ATB days, median, Q1-Q3) | P value |
| --- | --- | --- | --- | --- |
| **Per-protocol**  **SAPS-3 ≤ 59**  **Community-acquired infection**  **Lower respiratory tract infection**  **Appropriate empirical therapy** | 118  7 (5-9)  66  7 (5 – 8)  56  7 (5 – 7)  58  7 (5 – 8)  117  7 (5 – 8) | 59  6 (5 – 8)  30  6 (5 – 7)  25  6 (5 – 7)  27  6 (5 – 7)  58  6 (5 – 7) | 59  7 (7 – 10)  36  7 (7 – 10.5)  31  7 (6 – 8)  31  7 (7 – 8)  59  7 (7 – 9) | **0.011**  **0.003**  **0.008**  **0.032**  **0.009** |

ATB, Antibiotic therapy; CRP, C reactive protein; SAPS-3, Simplified Acute Physiology Score 3.
